# Molecular Modeling to Estimate the Diffusion Coefficients of Drugs and Other Small Molecules

**DOI:** 10.3390/molecules25225340

**Published:** 2020-11-16

**Authors:** Shuichi Miyamoto, Kazumi Shimono

**Affiliations:** Faculty of Pharmaceutical Sciences, Sojo University, 4-22-1 Ikeda, Nishi-ku, Kumamoto 860-0082, Japan; kshimono@ph.sojo-u.ac.jp

**Keywords:** diffusion coefficient, molecular radius, molecular modeling, Stokes-Einstein equation

## Abstract

Diffusion is a spontaneous process and one of the physicochemical phenomena responsible for molecular transport, the rate of which is governed mainly by the diffusion coefficient; however, few coefficients are available because the measurement of diffusion rates is not straightforward. The translational diffusion coefficient is related by the Stokes–Einstein equation to the approximate radius of the diffusing molecule. Therefore, the stable conformations of small molecules were first calculated by molecular modeling. A simple radius *r*_s_ and an effective radius *r*_e_ were then proposed and estimated using the stable conformers with the van der Waals radii of atoms. The diffusion coefficients were finally calculated with the Stokes–Einstein equation. The results showed that, for the molecules with strong hydration ability, the diffusion coefficients are best given by *r*_e_ and for other compounds, *r*_s_ provided the best coefficients, with a reasonably small deviation of ~0.3 × 10^−6^ cm^2^/s from the experimental data. This demonstrates the effectiveness of the theoretical estimation approach, suggesting that diffusion coefficients have potential use as an additional molecular property in drug screening.

## 1. Introduction

Diffusion is a spontaneous process and one of the physicochemical phenomena by which a substance is distributed and as such, it plays an important role in the life sciences [1,2]. For example, following the administration of a pharmaceutical, drug molecules are transported via the bloodstream and distributed to organs by active and passive transport. As diffusion is the driving force behind passive transport [2,3], physicochemical information on drug diffusion is useful for analyzing drug delivery systems and pharmacokinetics as well as investigating the distributions or diffusion velocities of central nervous system drugs in the brain after they penetrate the blood–brain barrier [4,5,6]. With respect to drug discovery from bacteria, a multidimensional diffusion-based gradient culture system for bacteria has been recently devised [7]. This circular culture apparatus represents one application of diffusion in the life sciences. In relation to this culture system, we have more recently developed a method to estimate translational diffusion coefficients (hereinafter referred to as diffusion coefficients) of small molecules using diffusion experiments in agar gel [8]. To our knowledge, few experimental values of diffusion coefficients of small molecules have been reported, and one possible reason for this is that the diffusion coefficients are measured with a special apparatus [9,10] although rotational diffusion coefficients of macromolecules have been examined by the NMR experiments [11,12]. To be used in drug screening, however, we need quicker procedures that do not require special apparatus; in other words, computational approaches such as molecular modeling, for estimating diffusion coefficients of small molecules is desirable.

The mean-square travel distance of a particle diffusing in one dimension (*x*) is given by the Einstein–Smoluchowski equation:

(1) $$\bar{x^{2}}=2Dt$$

where *D* is the diffusion coefficient of the molecule (particle) and *t* is the length of time the molecule has been diffusing [13]. This equation shows that the magnitude of the diffusion coefficient governs the degree to which the molecule diffuses. When a diffusing molecule is approximated by a sphere of radius *r*, *D* is expressed by the Stokes–Einstein equation:

(2) $$D=\frac{k_{B}T}{6\pi r\eta_{0}}$$

where *k*_B_ is the Boltzmann constant, *η*_0_ is the solvent viscosity, and *T* is the absolute temperature [14]. Using this equation, the diffusion coefficient *D* can be determined from an approximated molecular radius.

The Stokes–Einstein equation has been used in previous studies to estimate the diffusion coefficients. As for small molecules, the diffusion coefficients have been estimated based on physical models such as space-filling models [15]. With respect to macromolecules, the rotational diffusion tensors and other hydrodynamic properties of the globular proteins with atomistic structures were calculated by means of the bead and shell computer models in conjunction with the NMR experiments [11,12] while some groups used the mesoscale coarse-grained models to estimate the translational diffusion coefficients, treating entire macromolecules as single interacting centers [16]. As far as we know, however, methods that use a series of stable conformers of small molecules derived by molecular modeling have not been reported to calculate diffusion coefficients by the Stokes–Einstein equation.

Therefore, we aimed to develop a method to theoretically estimate the diffusion coefficients of small molecules, such as sugars and drugs, based on the Stokes–Einstein equation. In our approach, the stable conformations of small molecules are first calculated using molecular modeling. The approximate radii of the conformers are then estimated, and the diffusion coefficients are finally calculated using the Stokes–Einstein equation. The goal of this work is to demonstrate the effectiveness of this approach by comparing the theoretically derived diffusion coefficients with the experimental diffusion coefficients reported in the literature. Increased availability of diffusion coefficients is expected to provide an additional physicochemical molecular descriptor to be used in drug screening.

## 2. Methods

**Figure 1:**
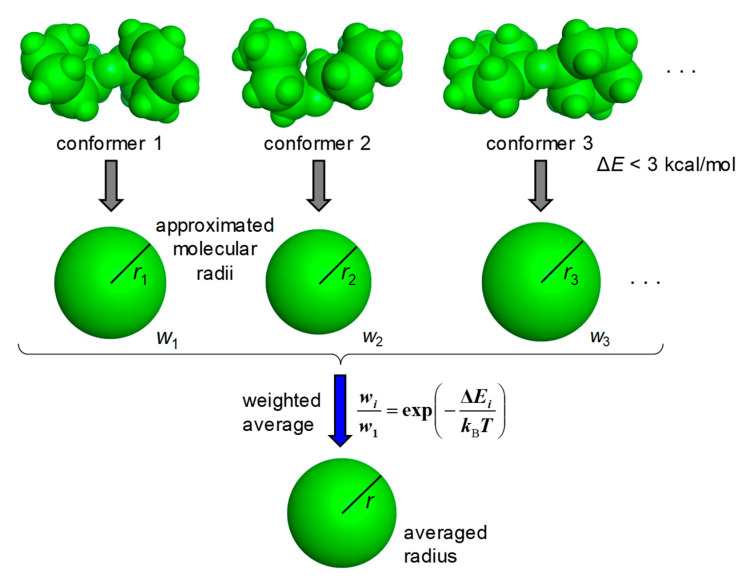
Derivation of the average radius of a molecule. Molecular radii are approximated for the stable conformations with Δ*E* < 3 kcal/mol, and the average radius is then derived as a weighted average of those. Based on Δ*E*_i_, the weight (*w*_i_) is calculated by the Boltzmann distribution at a temperature of 298 K.

The MOE (Molecular Operating Environment) software system developed by the Chemical Computing Group was used for the molecular modeling [17]. The carboxy group and the amino group were treated as the free form and the protonated form, respectively. The stable conformations of molecules were calculated using the Low Mode MD module with the force field MMFF94x [18]. Approximated molecular radii were calculated for the stable conformations with Δ*E* < 3 kcal/mol from the most stable conformation and the average radius was then derived by taking the Boltzmann distribution based on Δ*E* into account (Figure 1). The threshold of 3 kcal/mol was chosen because the contribution of the conformer with Δ*E* > 3 kcal/mol at 298 K was less than 1% compared with the most stable conformer.

**Figure 2:**
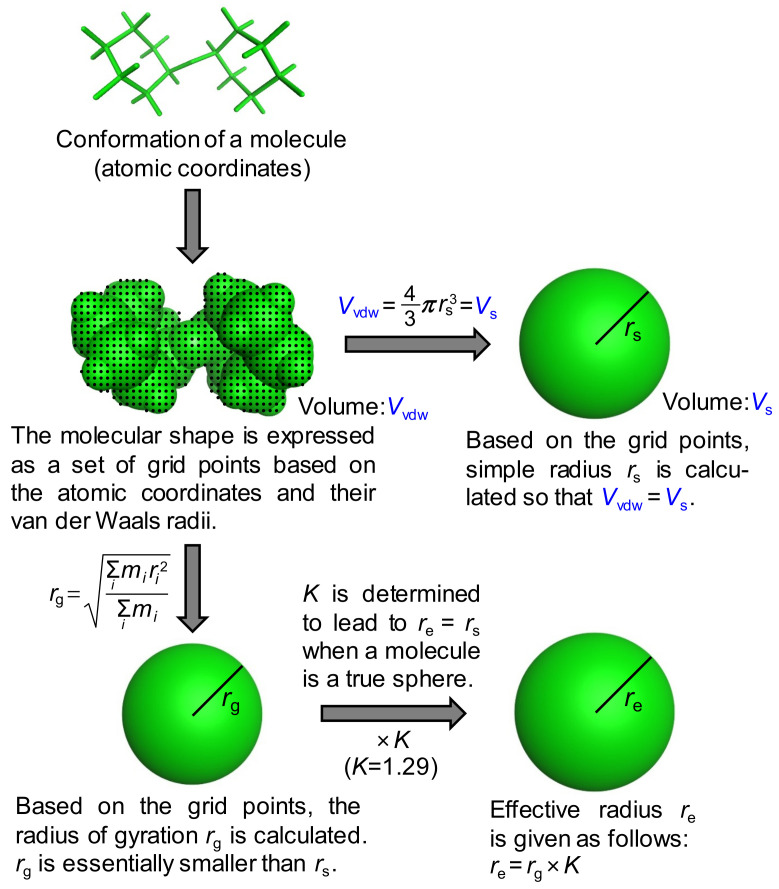
Derivation of the simple radius *r*_s_ and the effective radius *r*_e_, of a molecule. In the case of the radius of gyration *r*_g_, the mass is treated as evenly distributed in the molecule.

Although the concept of the approximated molecular radius is beyond this discussion, the specific procedure to calculate it is not trivial. Previously, the approximated radii were usually calculated based on the van der Waals volume of an ellipsoidal molecular shape, leading to some reduction for molecules having a radius less than around 4.5 Å [15] or the introduction of correction factors to individual molecules to account for the hydration effect [19]. The van der Waals volumes used were, however, calculated by the simple incremental rules of atoms or physical models such as space-filling models [20], indicating less accurate estimations of molecular volumes. As we used the latest molecular modeling technique, no correction factors were applied in advance. Two types of approximated radii, that is, a simple radius *r*_s_ and an effective radius *r*_e_, were thus proposed and calculated in this study (Figure 2).

In both cases, the molecular shape was first expressed as a set of grid points based on the atomic coordinates and their van der Waals radii. The volume within this shape is called the van der Waals volume (*V*_vdw_). The simple radius was derived using the following equation:

(3) $$V_{\mathrm{vdw}}=\frac{4}{3}\pi \;r_{s}^{3}=V_{s}$$

where *V*_s_. is the volume of a sphere with the simple radius *r*_s_. The effective radius was introduced based on the radius of gyration *r*_g_ to take into account the molecular shape. Based on the grid points, *r*_g_ is calculated by the following equation:

(4) $$r_{g}=\sqrt{\frac{\sum_{i}m_{i}r_{i}^{2}}{\sum_{i}m_{i}}}$$

where *i* represents the individual grid point and the mass is treated as evenly distributed among all points. The radius of gyration is essentially smaller than the simple radius and as for a generic sphere with the radius *r*, *r*_g_ is written as:

(5) $$r_{g}=\sqrt{\frac{3}{5}}r$$

The radius of gyration was then multiplied by the coefficient *K* to provide *r*_e_. In other words, the effective radius *r*_e_ is written as:

(6) $$r_{e}=\sqrt{\frac{5}{3}}r_{g}=1.29\;r_{g}=Kr_{g}$$

The value of 1.29, which converts the radius of gyration into the effective radius, was thus used for *K* in this work. Notably, a similar correction factor of ~1.3 was presented to compensate for the hydration shell effect by the Trovato group [16]. Diffusion coefficients *D*_s_ and *D*_e_ in water with *η*_0_ = 0.8902 mN·s/m^2^ were calculated for *r*_s_ and *r*_e_ at 298 K, respectively, using the Stokes–Einstein equation.

## 3. Results and Discussion

Diffusion coefficients were estimated for 18 compounds consisting of the sugars, amino acids, and drugs listed in Table 1. Xylose, fructose, galactose, and glucose are monosaccharides, and sucrose, lactose, trehalose, and maltose are heterodisaccharides. When dissolved in water, glucose exists as an equilibrated mixture of two different forms, α-d-glucose (α-form) and β-d-glucose (β-form). The ratio of α-form:β-form is known to be 36:64 [21]. The stable conformations of both forms of glucose were then investigated and the average *r*_s_ and *r*_e_ were calculated using the known ratio. Aspirin (CAS No. 50-78-2) and loxoprofen (CAS No. 68767-14-6) are non-steroidal anti-inflammatory drugs, and salbutamol (CAS No. 18559-94-9) is used to treat asthma. As for loxoprofen, two kinds of stereoisomers, specifically (2S, 2′R)-isomer and (2S, 2′S)-isomer were investigated. Fast Green FCF (CAS No. 2353-45-9) is a food coloring and was used in the agar-gel diffusion experiments [8]. Since its molecular weight corresponds to the upper limit of the drug candidates [22,23], it was consequently included and expedientially classified as a drug here.

Table 1 shows the number of conformers with Δ*E* < 3 kcal/mol, in addition to approximated radii and diffusion coefficients together with some literature data. The relative energies and Boltzmann populations as well as the cartesian coordinates of all the conformers of each molecule listed in Table 1 are found in the Supplementary Tables S1–S18 and MolFiles. Due to the extent of the flexibility of disaccharides, the numbers of disaccharide conformers with Δ*E* < 3 kcal/mol are generally greater than those of other conformers except for Fast Green FCF. According to the Stokes–Einstein equation, the diffusion coefficient of a molecule is expected to increase in inverse proportion to its approximate radius. Therefore, a larger molecular weight is related to a larger approximate radius, so that the diffusion coefficient is generally smaller. This broad trend is readily discernible in the data presented here. From Table 1, the simple radius *r*_s_ is smaller than the effective radius *r*_e_ in each case, and the diffusion coefficient *D*_s_ is accordingly larger than the diffusion coefficient *D*_e_, with the ratio of *r*_e_ to *r*_s_; in other words, the ratio of *D*_s_ to *D*_e_ ranges from 1.1 to 1.3.

*D*_0_ is the diffusion coefficient extrapolated to the infinitely dilute solution based on the diffusion coefficients obtained from solutions with different concentrations, *D*_c_. *D*_c_ for finite concentration solutions is generally observed to be smaller than *D*_0_ with no intersolute interactions. No intermolecular interactions are taken into account in the modeling; therefore, *D*_0_ corresponds to those (*D*_s_ or *D*_e_) derived by our method. As *D*_0_ is only reported in the literature for sugars, their *D*_0_ was first compared with the corresponding *D*_s_ and *D*_e_. Scrutiny of Table 1 reveals that *D*_0_ is between *D*_s_ and *D*_e_, and *D*_e_ showed good correspondence to *D*_0_ in most cases, with a reasonably small deviation of 0.27 × 10^−6^ cm^2^/s between them on average. Since the experimental standard deviation errors on the diffusion coefficients of sugars were around 0.2–0.3 × 10^−6^ cm^2^/s [10], our value of 0.27 × 10^−6^ cm^2^/s which correspond to the standard deviation error of 0.19 × 10^−6^ cm^2^/s, was comparable to previous experiments. The error for lactose was rather large among sugars but the reason for this is not obvious. Lactose has a β-glycosidic bond whereas the other disaccharides have α-glycosidic linkages and this might be related to the larger deviation. As for fructose, glucose, and sucrose, *D*_e_ was fairly consistent with *D*_0_ reported in the literature. These results suggest that the effective radius *r*_e_ is suitable for estimating the diffusion coefficients of sugars.

Next, the diffusion coefficients of the other compounds, that is, amino acids and drugs, are discussed. As observed from the sugar data in Table 1, *D*_c_ is smaller than *D*_0_, with an average deviation of 0.65 × 10^−6^ cm^2^/s. Assuming that a similar shift occurs for the other molecules, *D*_0_ for those molecules is derived by adding 0.65 × 10^−6^ cm^2^/s to *D*_c_ and are listed as _drv_*D*_0_ with parentheses in Table 1. In all cases, *D*_s_ calculated from *r*_s_ showed better correspondence to *D*_0_ with an average deviation of 0.30 × 10^−6^ cm^2^/s. As experimental concentrations of sugars were around five times higher than those of the other compounds, the actual *D*_0_ should be somewhat smaller than _drv_*D*_0_ in Table 1. Considering this, it is imagined that the average error of those approaches that of sugars. The error for alanine, with a molecular weight <100 as well as *r*_s_ < 3 Å, was larger than that of other molecules. According to the previous work, the diffusion coefficients of molecules with a small radius of around 2.7 Å would be corrected by multiplying ~1.2 [15]. In our case of alanine, the multiplication of *D*_s_ (9.01) by 1.1 provided good agreement with *D*_0_ (9.86), indicating a smaller correction factor. Such a correction, however, does not seem to be necessary to other molecules with *r*_s_ < 4.5 Å although it has been previously suggested [15]. Excluding alanine, the rather small average deviation of 0.24 × 10^−6^ cm^2^/s was obtained.

**Figure 3:**
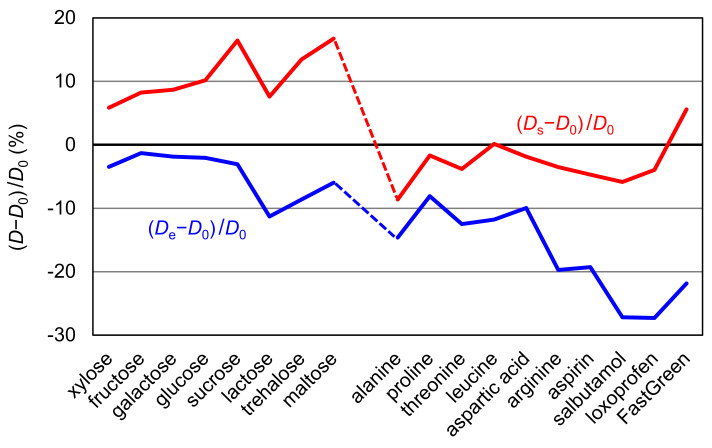
Relative deviations (%) of (*D*_s_ − *D*_0_)*/D*_0_ and (*D*_e_ − *D*_0_)*/D*_0_ were graphically represented for all molecules.

In Table 1, the error values of the estimated diffusion coefficients are presented as *D*_e_ − *D*_0_ and *D*_s_ − *D*_0_ for all compounds, and their percentage errors are graphically represented in Figure 3. From the figure, it is easily confirmed that *D*_e_ provided better correspondence to *D*_0_ for sugars whereas *D*_s_ showed better correspondence to *D*_0_. It also shows that the estimated values are smaller than *D*_0_ in most cases. When 0.25 × 10^−6^ cm^2^/s is then subtracted from the estimated values, a very small average deviation of 0.17 × 10^−6^ cm^2^/s is attained. Nonetheless, this procedure has no theoretical basis and must be regarded as being tentative. Further work may be required to elucidate the nature of this deviation.

From the above discussion, it may be said that the diffusion coefficients estimated by molecular modeling corresponded reasonably well with the experimental data. With respect to the two parameters for radii, *r*_e_ is suitable for sugars and *r*_s_ is suitable for the other compounds to be used with the Stokes–Einstein equation. The strong hydration ability of sugars seems to be related to this distinction due to the apparent effect of its size increase. From a different point of view, *r*_e_ may reflect the hydration effect properly, similarly to the correction factor for hydration used in the simplified macromolecular models [16]. This analysis has led to the conclusion that *r*_e_ should be used for molecules with strong hydration ability and *r*_s_ should be used for other compounds, providing the diffusion coefficients are accurate within the reasonable deviation of ~0.3 × 10^−6^ cm^2^/s.

In future work, we would like to increase the number and variety of compounds to answer questions such as: does a more favorable approximated molecular radius depend on the type of compound? Is there any single approximated molecular radius that reproduces the measured diffusion coefficients? Furthermore, refinement of the stable conformation with firmly hydrated water molecules may be achieved through molecular dynamics simulations in the bulk water model. Although such issues remain to be addressed currently, we consider that these results have some degree of positive significance because they demonstrate that diffusion coefficients can be reasonably estimated using a computational approach with molecular modeling. In drug screening, it is advisable to have enough physicochemical molecular descriptors such as P*k*_a_ and clogP [22]. As stated in Section 1, the diffusion velocities of central nervous system drugs might affect their efficacy. We hope our approach presented here will add the diffusion coefficient as one of those descriptors used for that purpose.

## 4. Conclusions

The stable conformations of small molecules, including drugs, were calculated using molecular modeling. Then, these conformations were used to propose and compute two types of approximate radii, *r*_s_ and *r*_e_. For molecules with strong hydration ability, the diffusion coefficients are best given by *r*_e_ from the Stokes–Einstein equation; for other compounds, the best diffusion coefficients are provided by *r*_s_, which has a reasonably small deviation of ~0.3 × 10^−6^ cm^2^/s from the experimental data. These results demonstrate the effectiveness of this computational approach and suggest its possible use as an additional molecular property in drug screening.

## Figures and Tables

**Table 1:** Approximated radii and diffusion coefficients of small molecules.

Molecule	MW ^b^	NoC ^c^	Radius (Å)		Diffusion Coefficient (×10^6^ cm^2^/s)					
					Estimated		Literature		Deviation	
			*r*_s_	*r*_e_	*D*_s_	*D*_e_	*D*_0_^d^	*D*_C_^i^	*D*_s_ − *D*_0_	*D*_e_ − *D*_0_
xylose	150	14	3.09	3.39	7.94	7.24	7.50 ^e^	6.78	0.44	−0.26
fructose	180	4	3.27	3.59	7.50	6.84	6.93 ^f^	6.63	0.57	−0.09
galactose	180	6	3.27	3.62	7.50	6.77	(6.90)	6.25	0.60	−0.13
glucose	180	10	3.28	3.69	7.48	6.65	6.79 ^g^	5.77	0.69	−0.14
sucrose	342	15	4.03	4.84	6.09	5.07	5.23 ^h^	4.93	0.86	−0.16
lactose	342	27	4.03	4.89	6.09	5.02	5.66 ^f^	4.59	0.43	−0.64
trehalose	342	10	4.04	5.04	6.07	4.89	(5.35)	4.70	0.72	−0.46
maltose	342	24	4.04	5.01	6.07	4.89	5.20 ^e^	4.71	0.87	−0.31
alanine	89	1	2.72	2.91	9.01	8.42	(9.86)	9.21	−0.85	−1.44
proline	115	2	2.97	3.18	8.25	7.71	(8.39)	7.74	−0.14	−0.68
threonine	119	2	2.95	3.24	8.31	7.56	(8.64)	7.99	−0.33	−1.08
leucine	131	7	3.20	3.51	7.66	6.75	(7.65)	7.00	0.01	−0.9
aspartic acid	133	2	2.92	3.19	8.39	7.70	(8.55)	7.90	−0.16	−0.85
arginine	174	2	3.41	4.10	7.19	5.98	(7.45)	6.80	−0.26	−1.47
aspirin	179	4	3.37	3.98	7.27	6.16	(7.63)	6.98	−0.36	−1.47
salbutamol	239	6	3.91	5.03	6.27	4.85	(6.66)	6.01	−0.39	−1.81
Loxoprofen ^a^	246	8 ^c^	3.89	5.17	6.30	4.77	(6.56)	5.91	−0.26	−1.79
Fast Green	763	30	5.41	7.30	4.54	3.36	(4.30)	3.65	0.24	−0.94

^a^ The (2S, 2′R)- and (2S, 2′S)-isomers of loxoprofen showed almost the same conformational and energy profile; results for the (2S, 2′R)-isomer are presented. ^b^ Molecular weight. The carboxy and amino groups were treated as the free and protonated forms, respectively. ^c^ Number of conformers with Δ*E* < 3 kcal/mol. ^d^ Values obtained by extrapolating from experimental data to an infinitely dilute solution. Values with parentheses represent _drv_*D*_0_ derived from *D*_c_. ^e^ Values taken from reference [24]. ^f^ The average value of 6.86 and 7.00 that were taken from references [10,25] is presented. ^g^ Values taken from Reference [10]. ^h^ Value taken from references [10,26]. ^i^ Values for sugars were taken from reference [8], and others were experimentally obtained by agar-gel diffusion experiments using the method described in reference [8].

## Supplementary Materials

The following are available online, Table S1: Relative energies and Boltzmann populations of stable conformers of xylose, Table S2: Relative energies and Boltzmann populations of stable conformers of fructose, Table S3: Relative energies and Boltzmann populations of stable conformers of galactose, Table S4: Relative energies and Boltzmann populations of stable conformers of glucose, Table S5: Relative energies and Boltzmann populations of stable conformers of sucrose, Table S6: Relative energies and Boltzmann populations of stable conformers of lactose, Table S7: Relative energies and Boltzmann populations of stable conformers of trehalose, Table S8: Relative energies and Boltzmann populations of stable conformers of maltose, Table S9: Relative energies and Boltzmann populations of stable conformers of alanine, Table S10: Relative energies and Boltzmann populations of stable conformers of proline, Table S11: Relative energies and Boltzmann populations of stable conformers of threonine, Table S12: Relative energies and Boltzmann populations of stable conformers of leucine, Table S13: Relative energies and Boltzmann populations of stable conformers of aspartic acid, Table S14: Relative energies and Boltzmann populations of stable conformers of arginine, Table S15: Relative energies and Boltzmann populations of stable conformers of aspirin, Table S16: Relative energies and Boltzmann populations of stable conformers of salbutamol, Table S17: Relative energies and Boltzmann populations of stable conformers of loxoprofen, and Table S18: Relative energies and Boltzmann populations of stable conformers of Fast Green FCF. MolFiles: The cartesian coordinates of all the conformers of each molecule listed in Table 1.
