# Supplementary material for: Molecular Modeling to Estimate the Diffusion Coefficients of Drugs and Other Small Molecules
Source: Molecules. 2020 Nov 16;25(22):5340. doi: 10.3390/molecules25225340 (PMC7709040; doi:10.3390/molecules25225340)
Supplement: Supplementary file 1 [file molecules-25-05340-s001.zip › SupplmntFiles/Sup.Tables/Table S1.docx]

**Table S1.** Relative energies and Boltzmann populations of stable conformers of xylose.

| **Entry No.** | **Δ*E*** **(kcal/mol)** | **Population ^1^** |
| --- | --- | --- |
| 1 | 0.00 | 1.000 |
| 2 | 0.22 | 0.690 |
| 3 | 0.45 | 0.468 |
| 4 | 0.99 | 0.188 |
| 5 | 1.23 | 0.123 |
| 6 | 1.41 | 0.093 |
| 7 | 1.51 | 0.078 |
| 8 | 1.78 | 0.051 |
| 9 | 2.38 | 0.018 |
| 10 | 2.62 | 0.012 |
| 11 | 2.72 | 0.010 |
| 12 | 2.75 | 0.010 |
| 13 | 2.93 | 0.007 |
| 14 | 2.94 | 0.007 |

^1^ Relative population is calculated by the Boltzmann distribution at a temperature of 298 K.
