# Supplementary material for: Molecular Modeling to Estimate the Diffusion Coefficients of Drugs and Other Small Molecules
Source: Molecules. 2020 Nov 16;25(22):5340. doi: 10.3390/molecules25225340 (PMC7709040; doi:10.3390/molecules25225340)
Supplement: Supplementary file 1 [file molecules-25-05340-s001.zip › SupplmntFiles/Sup.Tables/Table S18.docx]

**Table S18.** Relative energies and Boltzmann populations of stable conformers of Fast Green FCF.

| **Entry No.** | **Δ*E*** **(kcal/mol)** | **Population ^1^** |
| --- | --- | --- |
| 1 | 0.00 | 1.000 |
| 2 | 0.06 | 0.904 |
| 3 | 0.08 | 0.874 |
| 4 | 0.48 | 0.445 |
| 5 | 0.54 | 0.402 |
| 6 | 0.54 | 0.402 |
| 7 | 0.56 | 0.388 |
| 8 | 0.75 | 0.282 |
| 9 | 0.79 | 0.263 |
| 10 | 0.81 | 0.255 |
| 11 | 0.82 | 0.250 |
| 12 | 1.08 | 0.161 |
| 13 | 1.16 | 0.141 |
| 14 | 1.43 | 0.089 |
| 15 | 1.44 | 0.088 |
| 16 | 1.19 | 0.134 |
| 17 | 1.43 | 0.089 |
| 18 | 1.44 | 0.088 |
| 19 | 1.48 | 0.082 |
| 20 | 1.48 | 0.082 |
| 21 | 1.49 | 0.081 |
| 22 | 1.49 | 0.081 |
| 23 | 1.55 | 0.073 |
| 24 | 1.71 | 0.056 |
| 25 | 1.79 | 0.049 |
| 26 | 1.85 | 0.044 |
| 27 | 1.93 | 0.038 |
| 28 | 1.96 | 0.037 |
| 29 | 1.98 | 0.035 |
| 30 | 1.98 | 0.035 |

^1^ Relative population is calculated by the Boltzmann distribution at a temperature of 298 K.
