# Supplementary material for: Molecular Modeling to Estimate the Diffusion Coefficients of Drugs and Other Small Molecules
Source: Molecules. 2020 Nov 16;25(22):5340. doi: 10.3390/molecules25225340 (PMC7709040; doi:10.3390/molecules25225340)
Supplement: Supplementary file 1 [file molecules-25-05340-s001.zip › SupplmntFiles/Sup.Tables/Table S2.docx]

**Table S2.** Relative energies and Boltzmann populations of stable conformers of fructose.

| **Entry No.** | **Δ*E*** **(kcal/mol)** | **Population ^1^** |
| --- | --- | --- |
| 1 | 0.00 | 1.000 |
| 2 | 0.41 | 0.509 |
| 3 | 1.02 | 0.179 |
| 4 | 1.94 | 0.038 |

^1^ Relative population is calculated by the Boltzmann distribution at a temperature of 298 K.
