# Supplementary material for: Molecular Modeling to Estimate the Diffusion Coefficients of Drugs and Other Small Molecules
Source: Molecules. 2020 Nov 16;25(22):5340. doi: 10.3390/molecules25225340 (PMC7709040; doi:10.3390/molecules25225340)
Supplement: Supplementary file 1 [file molecules-25-05340-s001.zip › SupplmntFiles/Sup.Tables/Table S4.docx]

**Table S4.** Relative energies and Boltzmann populations of stable conformers of glucose.

| **Entry No.** | **Δ*E*** **(kcal/mol)** | **Population ^1^** |
| --- | --- | --- |
| α-1 | 0.00 | 1.000 |
| α-2 | 1.45 | 0.086 |
| α-3 | 1.58 | 0.069 |
| α-4 | 2.35 | 0.019 |
| α-5 | 2.43 | 0.017 |
| β-1 | 0.00 | 1.000 |
| β-2 | 1.44 | 0.088 |
| β-3 | 1.68 | 0.059 |
| β-4 | 2.15 | 0.026 |
| β-5 | 2.32 | 0.020 |

^1^ Relative population is calculated by the Boltzmann distribution at a temperature of 298 K.
