# Supplementary material for: Molecular Modeling to Estimate the Diffusion Coefficients of Drugs and Other Small Molecules
Source: Molecules. 2020 Nov 16;25(22):5340. doi: 10.3390/molecules25225340 (PMC7709040; doi:10.3390/molecules25225340)
Supplement: Supplementary file 1 [file molecules-25-05340-s001.zip › SupplmntFiles/Sup.Tables/Table S5.docx]

**Table S5.** Relative energies and Boltzmann populations of stable conformers of sucrose.

| **Entry No.** | **Δ*E*** **(kcal/mol)** | **Population ^1^** |
| --- | --- | --- |
| 1 | 0.00 | 1.000 |
| 2 | 0.66 | 0.329 |
| 3 | 0.82 | 0.252 |
| 4 | 0.98 | 0.192 |
| 5 | 1.01 | 0.181 |
| 6 | 1.30 | 0.111 |
| 7 | 1.86 | 0.044 |
| 8 | 1.95 | 0.037 |
| 9 | 2.12 | 0.025 |
| 10 | 2.47 | 0.015 |
| 11 | 2.48 | 0.015 |
| 12 | 2.53 | 0.014 |
| 13 | 2.58 | 0.013 |
| 14 | 2.73 | 0.010 |
| 15 | 2.95 | 0.007 |

^1^ Relative population is calculated by the Boltzmann distribution at a temperature of 298 K.
