# Supplementary material for: Molecular Modeling to Estimate the Diffusion Coefficients of Drugs and Other Small Molecules
Source: Molecules. 2020 Nov 16;25(22):5340. doi: 10.3390/molecules25225340 (PMC7709040; doi:10.3390/molecules25225340)
Supplement: Supplementary file 1 [file molecules-25-05340-s001.zip › SupplmntFiles/Sup.Tables/Table S6.docx]

**Table S6.** Relative energies and Boltzmann populations of stable conformers of lactose.

| **Entry No.** | **Δ*E*** **(kcal/mol)** | **Population ^1^** |
| --- | --- | --- |
| 1 | 0.00 | 1.000 |
| 2 | 0.23 | 0.674 |
| 3 | 0.25 | 0.659 |
| 4 | 0.30 | 0.602 |
| 5 | 0.48 | 0.430 |
| 6 | 0.50 | 0.430 |
| 7 | 0.54 | 0.404 |
| 8 | 1.10 | 0.134 |
| 9 | 1.20 | 0.132 |
| 10 | 1.25 | 0.121 |
| 11 | 1.38 | 0.100 |
| 12 | 1.39 | 0.098 |
| 13 | 1.50 | 0.080 |
| 14 | 2.02 | 0.034 |
| 15 | 2.07 | 0.031 |
| 16 | 2.25 | 0.024 |
| 17 | 2.35 | 0.019 |
| 18 | 2.39 | 0.016 |
| 19 | 2.44 | 0.016 |
| 20 | 2.47 | 0.015 |
| 21 | 2.64 | 0.012 |
| 22 | 2.64 | 0.011 |
| 23 | 2.73 | 0.010 |
| 24 | 2.85 | 0.008 |
| 25 | 2.88 | 0.008 |
| 26 | 2.95 | 0.007 |
| 27 | 2.98 | 0.007 |

^1^ Relative population is calculated by the Boltzmann distribution at a temperature of 298 K.
