# Supplementary material for: Molecular Modeling to Estimate the Diffusion Coefficients of Drugs and Other Small Molecules
Source: Molecules. 2020 Nov 16;25(22):5340. doi: 10.3390/molecules25225340 (PMC7709040; doi:10.3390/molecules25225340)
Supplement: Supplementary file 1 [file molecules-25-05340-s001.zip › SupplmntFiles/Sup.Tables/Table S7.docx]

**Table S7.** Relative energies and Boltzmann populations of stable conformers of trehalose.

| **Entry No.** | **Δ*E*** **(kcal/mol)** | **Population ^1^** |
| --- | --- | --- |
| 1 | 0.00 | 1.000 |
| 2 | 0.66 | 0.329 |
| 3 | 1.55 | 0.073 |
| 4 | 1.99 | 0.035 |
| 5 | 2.18 | 0.025 |
| 6 | 2.24 | 0.023 |
| 7 | 2.49 | 0.015 |
| 8 | 2.82 | 0.009 |
| 9 | 2.86 | 0.008 |
| 10 | 2.98 | 0.007 |

^1^ Relative population is calculated by the Boltzmann distribution at a temperature of 298 K.
