# Supplementary material for: Molecular Modeling to Estimate the Diffusion Coefficients of Drugs and Other Small Molecules
Source: Molecules. 2020 Nov 16;25(22):5340. doi: 10.3390/molecules25225340 (PMC7709040; doi:10.3390/molecules25225340)
Supplement: Supplementary file 1 [file molecules-25-05340-s001.zip › SupplmntFiles/Sup.Tables/Table S8.docx]

**Table S8.** Relative energies and Boltzmann populations of stable conformers of maltose.

| **Entry No.** | **Δ*E*** **(kcal/mol)** | **Population ^1^** |
| --- | --- | --- |
| 1 | 0.00 | 1.000 |
| 2 | 0.37 | 0.531 |
| 3 | 0.51 | 0.424 |
| 4 | 0.84 | 0.241 |
| 5 | 0.92 | 0.211 |
| 6 | 1.03 | 0.176 |
| 7 | 1.52 | 0.077 |
| 8 | 1.58 | 0.070 |
| 9 | 1.59 | 0.068 |
| 10 | 1.63 | 0.063 |
| 11 | 1.74 | 0.053 |
| 12 | 1.88 | 0.042 |
| 13 | 1.89 | 0.041 |
| 14 | 2.01 | 0.034 |
| 15 | 2.14 | 0.027 |
| 16 | 2.16 | 0.026 |
| 17 | 2.22 | 0.024 |
| 18 | 2.40 | 0.017 |
| 19 | 2.49 | 0.015 |
| 20 | 2.58 | 0.013 |
| 21 | 2.61 | 0.012 |
| 22 | 2.65 | 0.011 |
| 23 | 2.73 | 0.010 |
| 24 | 2.78 | 0.009 |

^1^ Relative population is calculated by the Boltzmann distribution at a temperature of 298 K.
